# Bacteriologic Enzymes as the Cause of Acquired Immunity

**Published:** 1899-10

**Authors:** 


BACTERIOLOGIC ENZYMES AS THE CAUSE OF ACQUIRED IMMUNITY.—"The substance resembling an enzyme, secreted by bacilli, not only destroys its own bacilli in time, but is also bactericidal to certain other species and neutralizes their toxins." Experiments with the enzyme derived from the bacillus pyocyaneus—"pyocyanose"—proved that not a single culture would develop after two hours, out of nearly five millions of anthrax bacilli to which the pyocyanose had been added *in vitro*. Tests on animals were still more conclusive; pyocyanose given with over a fatal dose of anthrax cultures or five hours later, saved the animals and no bacilli could be found in the blood.—*Journal American Medical Association.*

